# Experimental dataset for loads on hard rock shotcrete tunnel linings in a laboratory environment

**DOI:** 10.1016/j.dib.2024.110920

**Published:** 2024-09-10

**Authors:** August Jansson, Ignasi Fernandez, Carlos Gil Berrocal, Rasmus Rempling

**Affiliations:** a Chalmers University of Technology, Chalmersplatsen 4, 412 96 Göteborg, Sweden; b NCC, Drakegatan 10, 412 50 Göteborg, Sweden

**Keywords:** Distributed optical fiber sensors, Digital image correlation, Block load in tunnels, Distributed load in tunnels

## Abstract

To improve the understanding of failure mechanisms and behaviour of hard rock tunnel linings, local load conditions were experimentally simulated and monitored using a comprehensive set of sensors and imaging techniques. The data includes measurements from distributed optical fiber sensors (DOFS), high-resolution cameras, load cells, pressure cells and LVDTs. Two types of loads were examined: rock block load and bond loss combined with a distributed load over the area of lost bond. The experiments replicated these conditions and were conducted in a laboratory setting where the shotcrete and substrate rock were substituted by cast fiber reinforced concrete (FRC) and cast concrete, respectively.

To facilitate the loads, concrete cones were cast into the substrate concrete and pushed through the FRC top layer with a hydraulic jack to mimic rock block loads. To simulate the bond loss and the associated distributed load, lifting bags were installed and inflated between the FRC layer and substrate cast concrete. All specimens were monitored using DOFS embedded in two perpendicular directions and in two layers in the top FRC layer. In addition, the hydraulic jack was instrumented with LVDTs and load cells to measure displacement and load, and the pressure in the lifting bags was monitored using a pressure cell. Two cameras continuously photographed the top surface of the FRC layer, which had been painted with a speckle pattern, during the testing and the pictures can be used for digital image correlation (DIC). Lastly, each specimen was scanned with a 3D scanner prior to and after testing of the specimen.

Specifications Table

**Table:** 

Subject	Civil and structural engineering.
Specific subject area	Strain measurements performed with distributed optical fiber sensors in laboratory simulated hard rock shotcrete tunnel linings.
Type of data	Table/text file Images.
Data collection	Strain data were recorded through distributed optical fiber sensing, using an Optical Distributed Sensor Interrogator 6108 from Luna inc. Pictures were taken with two Aramis 12 M cameras for detecting displacements using Digital Image Correlation techniques. The software used to facilitate the calibration was GOM Correlate 2018. Force was measured using load cells and ring load cells. Pressure readings were taken from a preszsure gauge. Displacements were measured using LVDTs.
Data source location	*· Institution: Chalmers University of Technology* *· City/Town/Region: Göteborg* *· Country: Sweden*
Data accessibility	Repository name: Swedish National Data Service Data identification number: https://doi.org/10.5878/dvcn-bg03 Direct URL to data: https://snd.se/en/catalogue/dataset/preview/fddb7d3d-a442–476c-80c3–1b6aededc00a/1 Title: Experimental data for loads on tunnel linings including distributed optical fiber sensing and digital image correlation [1]
Related research article	None*.*

## 1 Value of the Data

• The experiments are designed to resemble loads acting on shotcrete tunnel linings in hard rock. Compared to earlier experiments [2,3], the load and load-bearing elements were applied in three directions instead of two.
• The dataset can be used to improve the understanding of failure mechanisms and behavior of shotcrete linings in hard rock tunnels [4].
• The data is beneficial to researchers and engineers working with design and assessment of shotcrete tunnel linings in hard rock.
• In future research, the data collected in this paper can be used to calibrate more accurate models for early identification of failures in shotcrete tunnel linings and to improve or validate existing design guidelines.

## 2 Background

The dataset presented in this article is the result of experiments carried out with the aim at characterizing strain patterns caused by loads from blocks or distributed loose rock acting on shotcrete tunnel linings. An exploratory full factorial experimental design was employed to investigate four factors varied within the specimens: (1) the adhesive bond between shotcrete and substrate, (2) the size of the loading area, (3) the type of loading applied, and (4) the thickness of the shotcrete layer. To ensure consistent properties of the substrate material, concrete slabs were cast to simulate the rock substrate material, and fiber reinforced cast concrete was used instead of fiber reinforced sprayed concrete (shotcrete), to enhance the precision during casting.

## 3 Data Description

All data files connected to these experiments are shared in the Swedish National Data Service (SND) public data repository. The files in the dataset consist of one compressed folder per specimen. Each compressed folder contains:

• Strain data from Distributed Optical Fiber Sensors (DOFS).
• Measurements from load cells, Linear Variable Differential Transformers (LVDT)s and pressure gauges.
• Images from two cameras for Digital Image Correlation (DIC) purposes.
• Point cloud data from 3D-scans of the specimen surface.

The data for each type of measurement is organized into separate folders within the compressed files. Additionally, material test data, including compressive cube tests, wedge splitting tests and tensile tests on drilled cores, are stored in a separate compressed file.

Strain data measured from the DOFS is stored as tabulated separated values files, “.tsv”-files. The initial 31 rows in all files contain metadata, such as the date and time for the measurement, sensor length, gauge length and sensor name. The 32nd row contains the tare-value for each gauge, representing the strain difference between the initial value obtained upon identification of the sensor and the strain value at the start of the measurement. The 33rd row lists all gauge positions along the sensor as length values from the start of the sensor in meters. Subsequent rows provide strain measurements for each point along the x-axis for every time step. The column position in the strain measurements corresponds to the column position of the x-axis. Similar “.tsv” - files ending with “-hp” contain measurements recorded during a “heat pinching” procedure, which is a method to locate the cable position in the specimen and further explained in the “Experimental design, materials and methods” section. The data in these files have the same structure as the other “.tsv” files and recorded strain peaks can be correlated to applied heat at certain points in the specimen.

Each compressed folder in the dataset also contains a file with data for load cells, pressure cells and LVDTs. The file format is “.dat” and the reading for each instrument is stored column wise. The specific instrument and unit are indicated in headers on row 4 and 5 respectively. For cone-loaded specimens, the 1st column contains load data in kN acquired from a load cell underneath the hydraulic jack. The 2nd column shows the displacement of the hydraulic jack in mm, and in columns 3–6, the load measured in ring load cells placed around steel rods are presented in kN. In the last column, 7, the time from the start of the experiments is given in seconds.

In the “.dat”-files for bag-loaded specimens, the 2nd column contains data for the displacement, in mm, of the hydraulic jack pushing oil into the bag, while the 3rd column includes data from the pressure cell connected to the lifting bag in kPa. Columns 4–6 contain data for the ring load cells around the steel rods in kN, and in the last column, 7, the time from the start of the test is presented in seconds.

For each specimen, two image series were taken with two separate cameras to facilitate Digital Image Correlation (DIC) in three directions. These images are stored in each specimen's corresponding compressed folder, sorted in the subfolder “DICImages”. The images are further sorted in subfolders named “cam00” and “cam01” and saved as “.tif”-files, numbered sequentially with image-000000.tif. The images were taken at 0.5 Hz frequency.

**Fig. 1:**
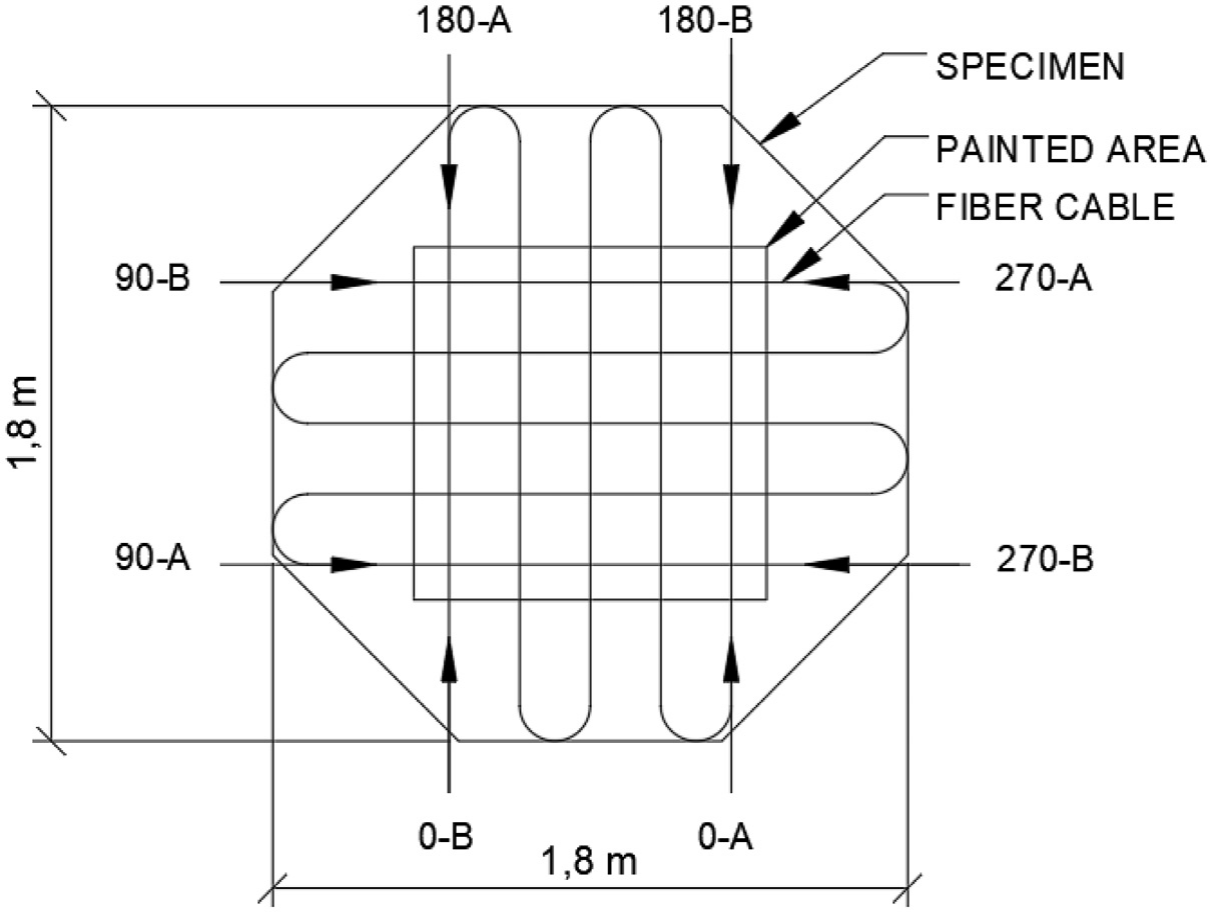
Possible starting positions and directions for the DOFS data.

To align the DOFS data and the photos taken for DIC, the initial direction of the DOFS is provided in a readme file stored with the sensor files. The direction is given in relation to the orientation of the DIC images, in which marks at 0, 90, 180 and 270° were drawn on the specimen surfaces. Possible directions of the fiber are shown in Fig. 1, and an example of the readme file content is shown in Table 1.

**Table 1:** Example of information in readme files belonging to distributed optical fiber sensor data.

Specimen ID	DOFS file name	DOFS Position file name	Start direction
CS50G	CS50G-1-top	CS50G-1-top-hp	0-B
CS50G	CS50G-1-bot	CS50G-1-bot-hp	0-B
CS50G	CS50G-2-top	CS50G-2-top-hp	90-B
CS50G	CS50G-2-bot	CS50G-2-bot-hp	90-B

The last data type in the specimen specific compressed folders is point cloud data acquired through 3D scanning. A scan was performed for all specimens before and after testing, and the point cloud data are stored as “.pts” in the “3DScanning" subfolder. The files can be read as text files and each row represents a point from the scan. Each point includes an x, y and z coordinate followed by an RGB value. The origin of the coordinates is determined from the first recorded point for each scan.

In the final compressed folder, “MaterialData.zip”, material test data related to compression tests for concrete cubes, tensile tests for drilled concrete cores and wedge splitting tests are stored. For the wedge splitting tests, the first column provides the crack mouth opening displacement (CMOD) in mm, and the total vertical displacement of the wedge is given in the 2nd column in mm. The applied load, measured by a load cell, is presented in the 3rd column, in kN, and the time from the start of the test is given in the 4th column. Similarly, for the cores tested in tension, the displacement, in mm, is given in the 1st column, followed by the load in kN in the second column, and the time from the start of the test in seconds is given in the last, 3rd column.

For the concrete cube compression tests, the file type format is “.TRA”, and the files consist of three columns representing machine displacement in mm, load applied in kN, and time in seconds from the start of the test, respectively. Metadata for date, specimen geometry, weight, and strength are given in the first 14 rows.

## 4 Experimental Design, Materials and Methods

### 4.1 Specimen geometry and preparation

The specimens used in this experimental campaign consisted of two layers of cast concrete. The bottom layer, representing a rock substrate, was cast as ordinary concrete with a strength grade of C35/45. The top layer was cast as fiber-reinforced concrete, using a mix composition similar to that of a typical shotcrete mix, given in Table 2. The substrate slabs were cast on two separate occasions, with eight slabs each time and a 7-day interval between them. The top layer was cast for all specimens at 63 and 56 days after the substrate slabs were cast, respectively. Additionally, in all specimens, four 50 mm diameter plastic tubes were symmetrically installed in a square formation with 1 m side lengths to allow for fastening of steel rods during the tests. All dimensions and parts of the slabs are shown in Fig. 3.

**Table 2:** Concrete recipe for top layer of fiber reinforced concrete.

Material	Type & supplier	kg/m3
Cement	CEM I 42,5N SR3 MH/LA (Norcem, Brevik)	490
0/6 fine aggregate	Crushed (Skanska Vikan)	540
0/4 fine aggregate	Natural (Kroghs A/S)	753
4/8 coarse aggregate	Crushed (Skanska Vikan)	275
Steel fiber	HE 55/35 GL (ArcelorMittal)	53
Air entrainer	Master air 1 (Master Bilder)	0,5 (0,10 % of C)
Retarder	MasterRoc (Master Bilder)	0,8 (0,16 % of C)
Super plasticizer	Master Glenimum 51/18 ((Master Bilder)	1,7 (0,34 % of C)
*Super plasticizer*	*Master Glenimum 51/18 ((Master Bilder) – at delivery*	*3,0*
Water		206
w/c-ratio		0,42 [-]

Two types of loading conditions were studied in this experimental campaign:

(1) A rock fallout simulated as a concrete cone pushed through the shotcrete layer using a hydraulic jack.
(2) A loss of adhesive bond between rock and shotcrete, combined with a distributed load caused by loose rock particles, simulated by an inflated lifting bag placed between the substrate and top concrete layers.

**Fig. 2:**
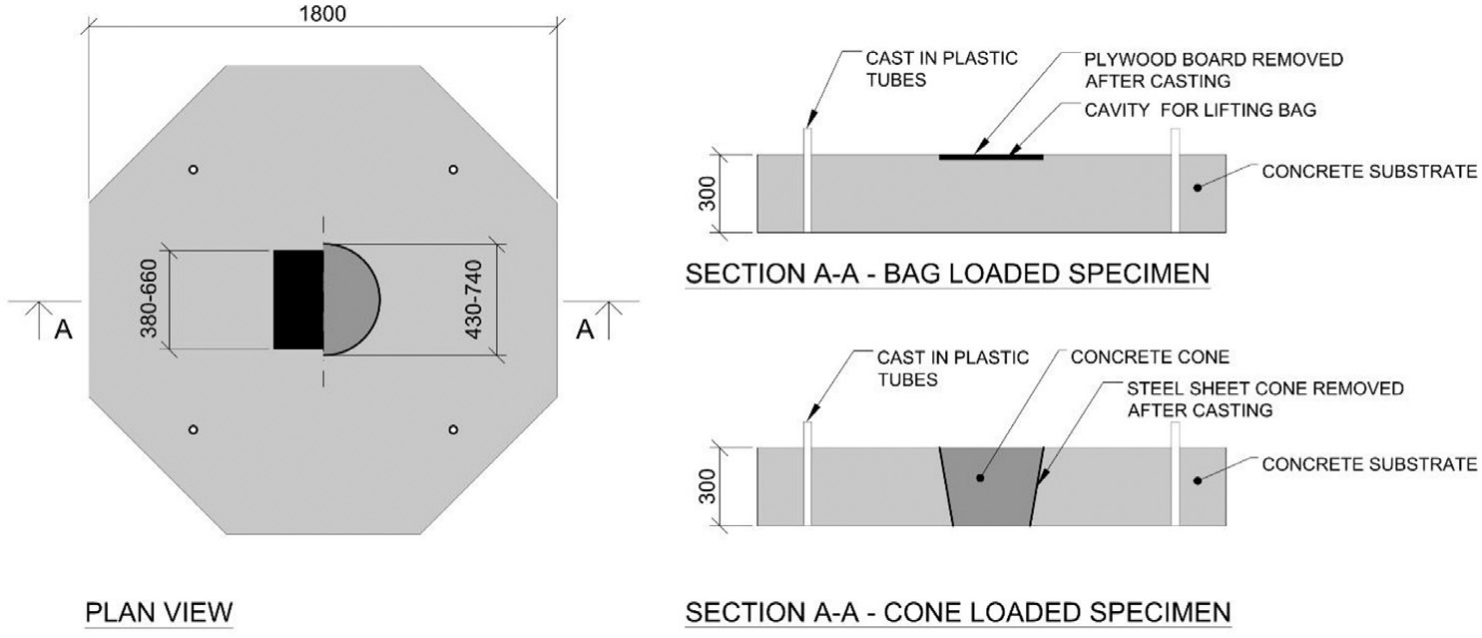
Illustration of casting details for the substrate slabs. Plywood boards were used to create a cavity for the lifting bags and steel sheets were used for casting the concrete cones.

The concrete cones were cast within the substrate slabs by attaching a steel sheet cone to the formwork's bottom before casting as shown in Fig. 2. Once the concrete had hardened, the concrete cones were lifted out of the substrate slabs and the steel sheet cones were removed. For the specimens with lifting bags, square plywood boards were used to create a cavity in the substrate concrete layer during casting, where the lifting bags were later placed, see Fig. 2. Furthermore, the edges of the lifting bags were sealed with sealing strips to prevent concrete from leaking into the cavity during the casting of the top concrete layer. Additionally, a plastic sheet was glued on top of the bags to ensure zero adhesive bond between the top layer concrete and the lifting bag. A tube was installed from a corner of the lifting bag to the side edge of the substrate concrete slab to enable pressurizing of the bag.

**Fig. 3:**
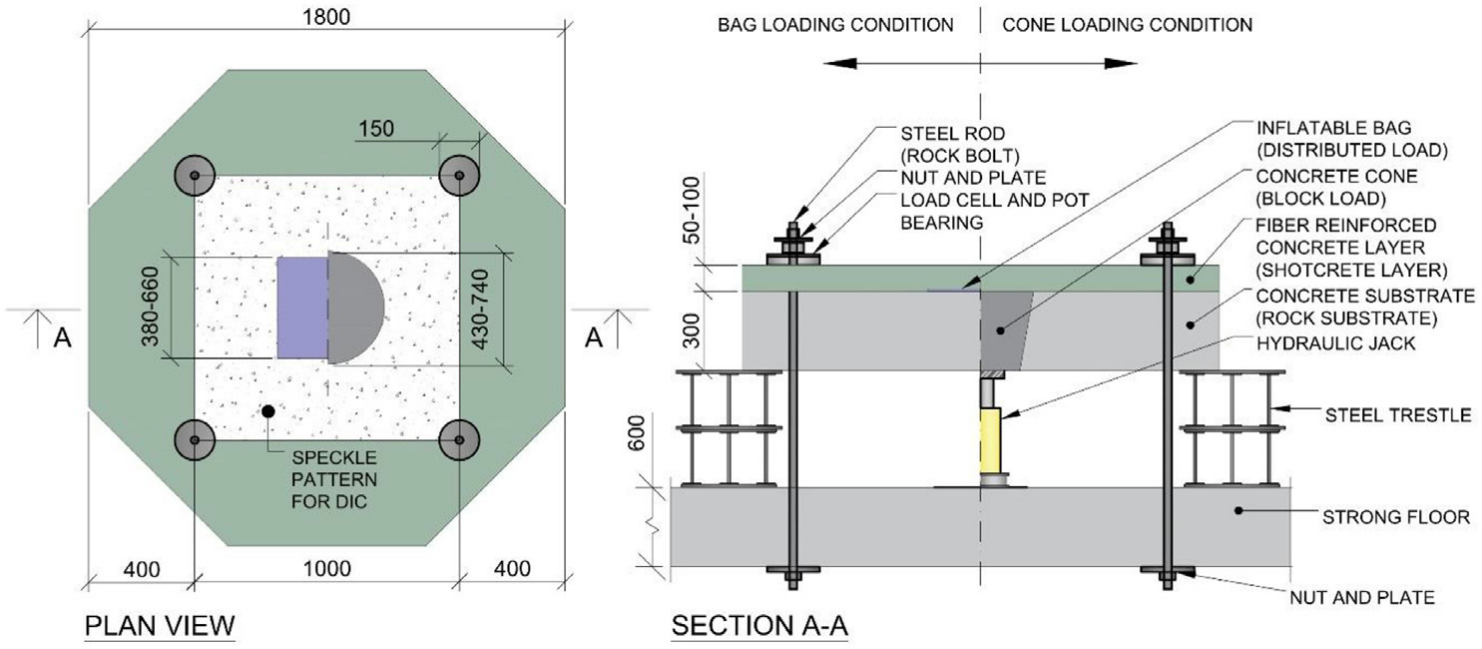
Dimensions and composition of experimental specimens. Text in parenthesis indicates the intended element of a shotcrete tunnel lining system.

Fig. 3 shows a schematic representation of the two loading conditions, as well as the different sizes of cones and bags used in the experiments. The large and small lifting bags used were of Trelleborg TLB 32 and Sava SLK 10 models, respectively.

To vary the adhesive bond between the substrate and top concrete layers, the surface of the substrate slabs was pre-treated by either grinding or hydro-demolition. Grinding was performed using an OPTIROC ABS8331 grinding machine, while hydro-demolition was carried out with a 0.7 mm porcelain nozzle positioned at 15–20 cm from the surface, operating at 2200 bar.

Table 3 lists all the varied factors in the experiments, along with the abbreviations used for each specimen.

**Table 3:** Factorial design of experiments and nomenclature for each specimen.

	Loading condition							
	Cone				Bag			
	Loading area				Loading area			
	Small		Large		Small		Large	
	Thickness		Thickness		Thickness		Thickness	
	50 mm	100 mm	50 mm	100 mm	50 mm	100 mm	50 mm	100 mm
**Ground**	CS50G	CS100G	CL50G	CL100G	BS50G	BS100G	BL50G	BL100G
**Hydro**	CS50H	CS100H	CL50H	CL100H	BS50H	BS100H	BL50H	BL100H

### 4.2 Mounting of optical fiber cables

**Fig. 4:**
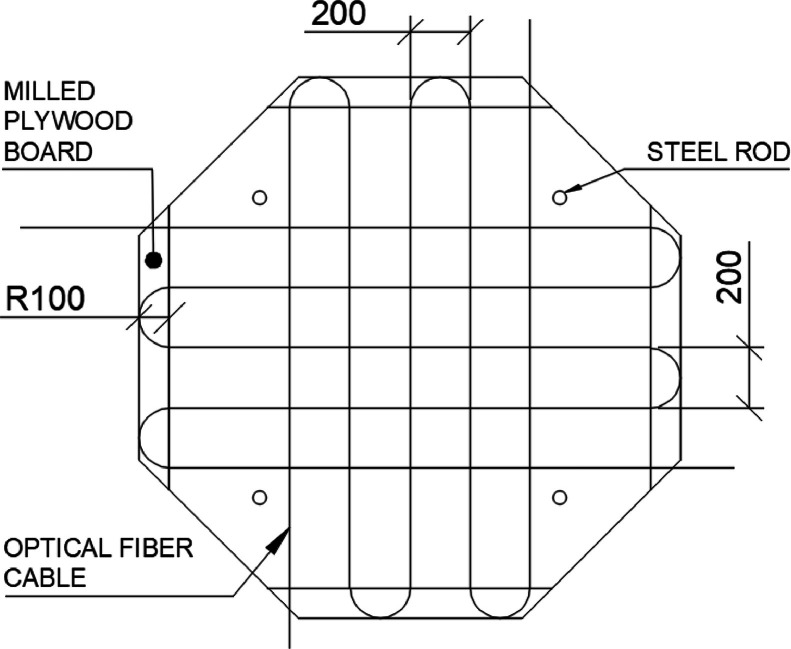
Arrangement of optical cables in one layer.

**Fig. 5:**
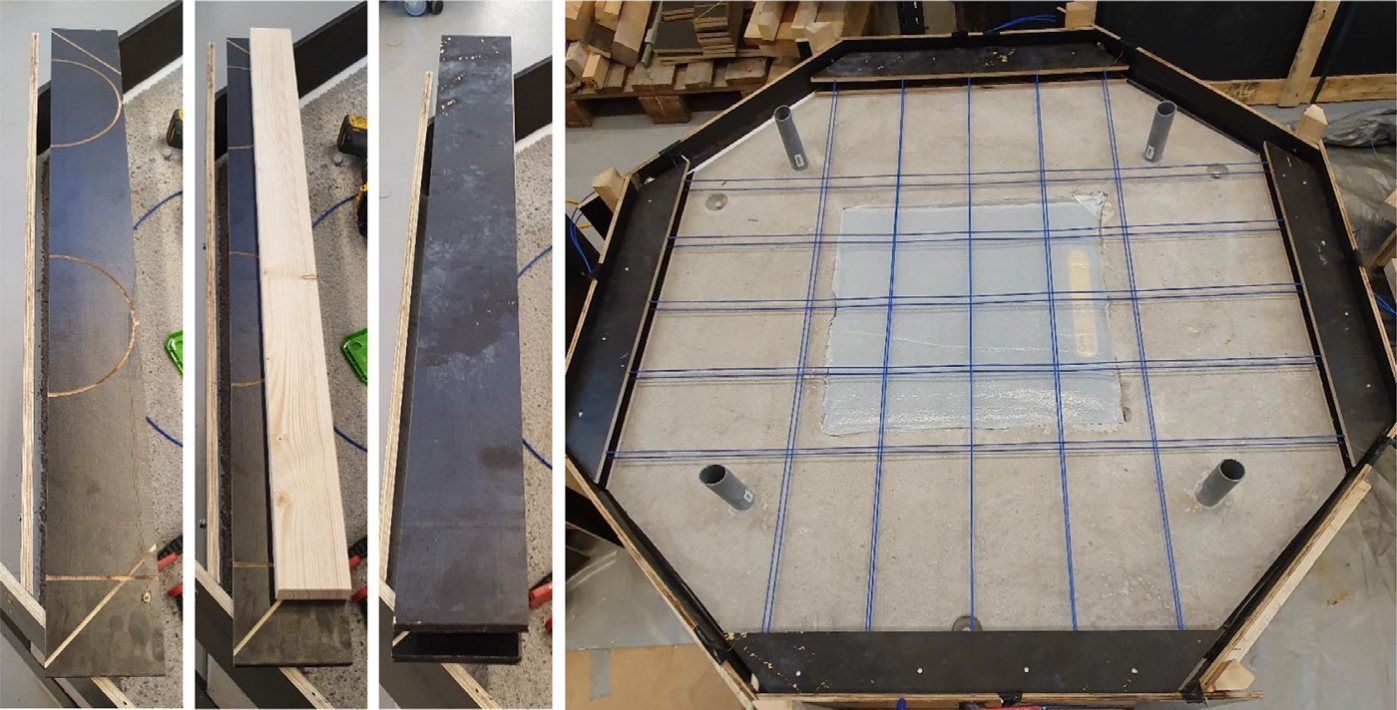
CNC-Milled plywood boards with slits for optical cables (left), and a specimen instrumented with optical fibers prior to the casting of the top concrete layer (right).

Distributed Optical Fiber Sensors (DOFS) were installed in the top fiber reinforced concrete layer. In each specimen, four BRUsens V9 optical fiber cables from Solifos were installed in a grid configuration consisting of two layers arranged in two orthogonal directions. The bottom layer of optical cables was positioned 10 mm above the substrate slab, while the top layer was placed 10 mm below the top surface of the specimen. Each cable was laid out in a serpentine pattern between two opposite edges of the specimens, with a spacing of 200 mm between each straight segment. Fig. 4 shows the layout of one of the layers of the optical fiber cables. Each optical fiber cable formed a total of 5 straight segments, resulting in 20 straight segments in each specimen. The turns in the serpentine pattern were facilitated by installing plywood boards with CNC-milled 100 mm diameter half-circular grooves to guide the cables. Fig. 5 shows the setup of plywood boards at one edge of a specimen as well as the finished layout of the optical cables prior to the casting of the top fiber reinforced concrete layer.

**Fig. 6:**
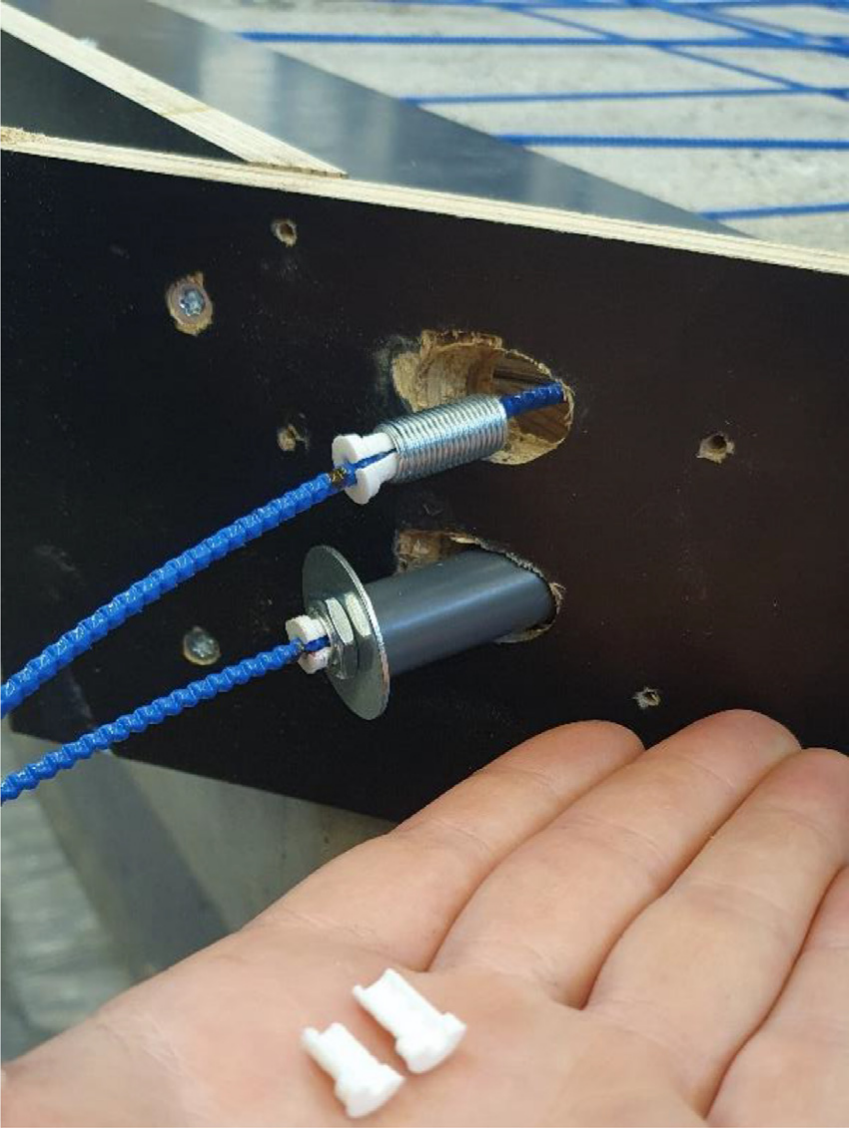
3D-Printed plugs (white) fixating the threaded cylinder that is used to tension the optical cable (blue) with the nut. The top cable shows how the cylinder is fixated, and the bottom shows how the nut is used to tension the cable in combination with a plastic tube and washer.

To secure the optical cables to the formwork, 3D-printed plugs matching the texture of the cables were utilized. The plugs were used to clamp an externally threaded cylinder to the cable, and by using a washer and a plastic tube as support to the formwork, the cable was tensioned as a nut was screwed onto the cylinder as depicted in Fig. 6.

### 4.3 Test setup

**Fig. 7:**
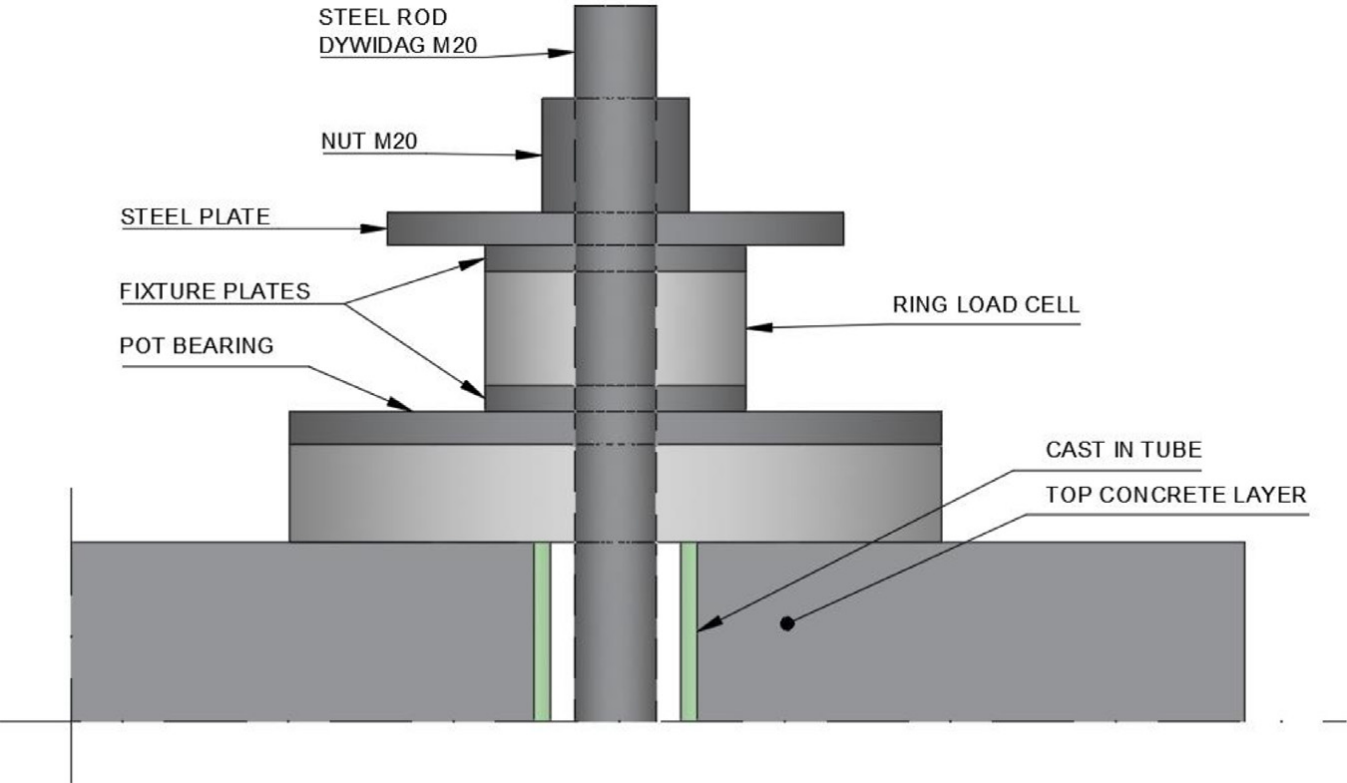
Detail figure for the fastening of Dywidag steel rods and placement of fixture plates for ring load cells and pot bearings.

The specimens were positioned on top of two steel trestles and secured to the strong floor using steel rods. Preexisting holes in the floor slab allowed the steel rods to be fastened with steel plates and nuts on both sides of the slab. The steel rods penetrated the specimens through the cast-in tubes and were fastened on the top surface of the specimens, as shown in Fig. 7. A pot bearing and a load cell were mounted and tightened with a steel plate and a nut on the top surface using a wrench and hand force. Additionally, a 1 m × 1 m region on the top surface of the specimens was painted with a black and white stochastic pattern for improving the contrast when applying DIC, and two cameras were mounted approximately 1.5 m above the specimens for digital image correlation measurements.

For the specimens subjected to the cone loading condition, a displacement-controlled hydraulic jack was used. The hydraulic jack was placed underneath the specimen, and loading was applied at an initial loading rate of 0.5 mm/min, which was increased to 2 mm/min after reaching the peak load. The relative displacement between the cones and the bottom slab was measured using three evenly spaced LVDTs glued to the bottom of the substrate slab. The load was measured by a load cell located underneath the hydraulic jack and by ring load cells around the steel rods. A measurement rate of 8 Hz was utilized for all load cells and LVDTs.

In the case of specimens with a cast-in lifting bag, hydraulic oil was used to fill and pressurize the bags. The hydraulic pump in the laboratory had a maximum capacity of 300 bars, while the lifting bags were rated for a maximum pressure of 8 bars. To mitigate the risk of over pressurization, a smaller cylindrical hydraulic jack with an area of 6.4 cm^2^ (Enerpac RC59) was used to push against a larger cylindrical hydraulic jack with an area of 133 cm^2^ (Larzep D10032). The larger hydraulic jack was connected to the lifting bag and filled it with oil, while the pressure was measured using a pressure cell installed between the jack and bag. During loading, the smaller jack was displacement controlled at 16 mm/min, resulting in 0.213 l of oil per minute into the bag. The ring load cells at the steel rods and the pressure cell connected to the lifting bag were measured at 8 Hz. The larger hydraulic jack had a maximum volume of 4.25 l, and the test was paused for refilling when all the oil had been pushed from the jack to the bag. For the large bags, the hydraulic jack was refilled three times, while two refills were required for the small bags. During the testing of the bag-loaded specimens, the small cylinder was replaced with a larger cylinder (Enerpac RC1010), with an area of 14.4 cm^2^ to increase the maximum pressure in the bag.

During each test, the fiber optic cables of the tested specimen were connected to an Optical Distributed Sensor Interrogator 6108 from Luna inc. that measured strains at 6.25 Hz with a gauge pitch of 5.2 mm. Prior to casting the top concrete layer, a loop of resistor wire was coiled around the optical cable at each outlet point of the milled plywood boards. All resistor wires were connected in series, and before loading the specimens, a current was applied through the wire to heat it up while strains were recorded from the optical cables to identify the position of the cables within the specimen. In the “Data description” section, this method is referred to as “heat pinching”. Furthermore, the top surface of the specimens was recorded using the two cameras (Aramis 12 M) installed above the specimens at 0.5 Hz.

### 4.4 Deviations in test setup

During testing, some specimens did not adhere to the procedures described above due to unexpected issues. For the first tested specimen, which involved the small cone loading condition with a 100 mm thick top layer and a hydro-demolished interface, the test was interrupted because one of the LVDTs between the substrate slab and cone ceased recording. After two attempts to restart the test, the issue was identified and resolved, allowing the test to be continued. Additionally, during the demounting of the final specimen with cone loading condition, one of the ring load cells broke due to large inclinations at the steel rods. As a result, only 3 ring load cells were used for the bag loaded specimens.

Tests involving the lifting bag loading condition encountered further complications. Firstly, the load capacity of two specimens exceeded the maximum load provided by the inflatable bags, preventing them from being loaded to failure. Secondly, the calibration of the pressure cell encountered technical issues, resulting in recordings of values 2.5 times higher than real values. The appended data has been adjusted for this and real pressures are presented. In addition, new valves and a larger hydraulic cylinder were installed to increase the capacity during the test runs.

Table 4 lists all the attempts and modifications made to the tests involving the lifting bag loading condition in chronological order. Highlighted in

**Table 4:** Summary of testing sequence for lifting bag loading condition specimens and updates in testing procedure. Bold attempt indexes indicate the final test run for the specimen. Attempts marked with (*) did not reach failure.

Specimen ID	Loading condition	Substrate surface	Top layer thickness	Attempt
1 (BS100G)	Small bag	Ground	100 mm	1
2 (BL50H)	Large bag	Hydro demolished	50 mm	**1**
3 (BL50G)	Large bag	Ground	50 mm	**1**
4 (BL100G)	Large bag	Ground	100 mm	1
**New valves**				
4 (BL100G)	Large bag	Ground	100 mm	**2**
5 (BL100H)	Large bag	Hydro demolished	100 mm	1
1 (BS100G)	Small bag	Ground	100 mm	2
**Pressure cell recalibration**				
1 (BS100G)	Small bag	Ground	100 mm	3
**Larger hydraulic jack**				
1 (BS100G)	Small bag	Ground	100 mm	4*
5 (BL100H)	Large bag	Hydro demolished	100 mm	**2**
6 (BS50G)	Small bag	Ground	50 mm	**1**
7 (BS50H)	Small bag	Hydro demolished	50 mm	**1**
8 (BS100H)	Small bag	Hydro demolished	100 mm	1*

Table 4 with an asterisk (*) are specimens with a small bag and a 100 mm thick top concrete layer (BS100H and BS100G), which did not reach failure as the maximum capacity of the system was reached.

### 4.5 Material testing

In addition to the tests described in the previous sections, material testing was conducted. For each casting of the substrate slabs, three concrete cubes (150 mm × 150 mm) were cast and stored under water until testing. During the casting session for the top fiber reinforced concrete layer, four cubes (150 mm × 150 mm) were cast and stored under water, as well as six wedge splitting test cubes as outlined in [5]. Furthermore, in each specimen with a lifting bag loading condition, a core was drilled close to an edge for uniaxial tensile tests as in [6]. The material tests were carried out one week after the main tests, at 101 and 108 days for the concrete cubes and at 45 days for the wedge splitting tests. All concrete cubes were tested in accordance to EN 12390-3:2019 and the wedge split tests according to [5].

## Limitations

The data collected during each test run was collected using three computers, one for loads, pressure and displacement, one for photos and one for the DOFS. The computers were not connected and, thus, threre was no time synchronization between the computers.

The load conditions used in the presented experimental design are intended to simulate loads in shotcrete tunnel linings. However, instead of shotcrete and rock substrate, cast concrete was used. Furthermore, a flat octagonal slab was used instead of an arching tunnel section due to size and weight limitations in the lab.

## Ethics Statement

The authors have read and followed the ethical requirements for publication in Data in Bried and confirm that the current work does not involve human subjects, animal experiments, or any data collected from social media platforms.

## CRediT Author Statement

**August Jansson:** Methodology, Writing – Original Draft **Ignasi Fernandez:** Conceptualization, Methodology, Supervision **Carlos Gil Berrocal:** Conceptualization, Writing – Reviewing and Editing, Supervision **Rasmus Rempling:** Conceptualization, Methodology, Supervision, Funding acquisition, Project administration.

## Data Availability

Experimental data for loads on tunnel linings including distributed optical fiber sensing and digital image correlation (Original data) (Swedish National Data Service).
